# Supplementary material for: Modification of the structural stability of human serum albumin in rheumatoid arthritis
Source: PLoS One. 2023 Mar 17;18(3):e0271008. doi: 10.1371/journal.pone.0271008 (PMC10022781; doi:10.1371/journal.pone.0271008)

**Structural stability of Human serum albumin is modified in rheumatoid arthritis (**Supplemental Figure and Table**)**

Hsien-Jung L. Lin^1^*, David H. Parkinson^1^*, J. Connor Holman^1^, W. Chad Thompson^1^, Christian N. K. Anderson^1^, Marcus Hadfield^1^, Stephen Ames^1^, Nathan R. Zuniga Pina ^1^, Jared N. Bowden^1^, Colette Quinn^2^, Lee D. Hansen^1^, John C. Price^1^

1. Department of Chemistry and Biochemistry, Brigham Young University, Provo, UT 84602 USA
2. TA Instruments, 860 W 410 N, Lindon, UT 84042 USA

* Authors contributed equally

# Supplemental Figure 1: All HDCs per group

**
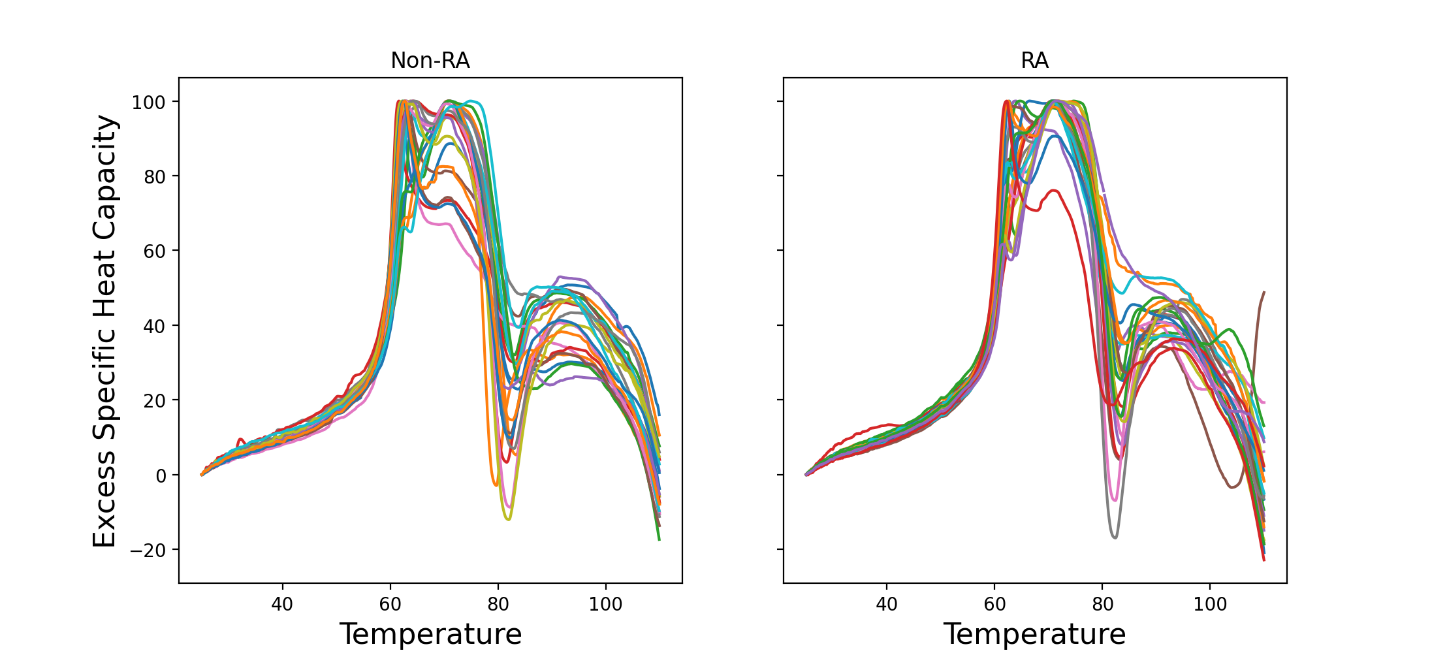
**Supplemental Figure 1 plots the normalized signal vs temperature from raw output. Plots are sorted by RA diagnosis.

# Supplemental Figure 2: Average HDCs with Deviation:


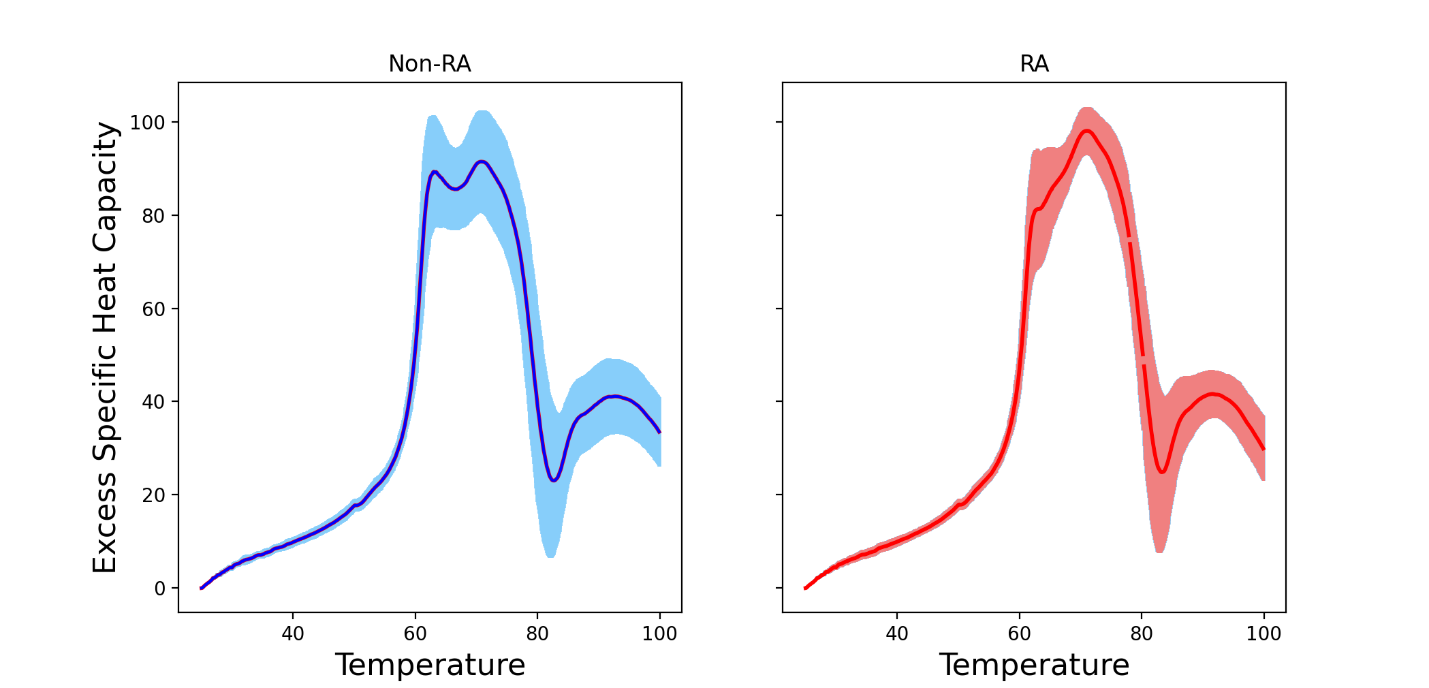


Supplemental Figure 2 shows the average HDC plot for RA and Non-RA samples, with the shaded region showing the standard deviation. These two figures are overlayed in Figure 3A.

# Supplemental Table 1: Protein Fold Changes

| . | RA vs. Non-RA | | | | | LPR vs. HPR | | | | |
| --- | --- | --- | --- | --- | --- | --- | --- | --- | --- | --- |
|  | **Protein Accession** | **Protein Name** | **Rank** | **Fold Change** | **p-Value** | **Protein Accession** | **Protein Name** | **Rank** | **Fold Change** | **p-Value** |
| Top 8 Proteins | P02768\|ALBU | Serum Albumin | 1 | 0.96 | 0.58 | P02768\|ALBU | Serum Albumin | 1 | 0.96 | 0.58 |
|  | P0DOX5\|IGG1 | IgG Variable 1 | 2 | 1.06 | 0.71 | P0DOX5\|IGG1 | IgG Variable 1 | 2 | 1.06 | 0.71 |
|  | P01857\|IGHG1 | IgG Heavy 1 | 3 | 1.05 | 0.77 | P01857\|IGHG1 | IgG Heavy 1 | 3 | 1.05 | 0.77 |
|  | P01859\|IGHG2 | IgG Heavy 2 | 4 | 1.09 | 0.56 | P01859\|IGHG2 | IgG Heavy 2 | 4 | 1.09 | 0.56 |
|  | P01860\|IGHG3 | IgG Heavy 3 | 5 | 1.09 | 0.55 | P01860\|IGHG3 | IgG Heavy 3 | 5 | 1.09 | 0.55 |
|  | P00738\|HPT | Haptoglobin | 6 | 0.88 | 0.48 | P00738\|HPT | Haptoglobin | 6 | 0.88 | 0.48 |
|  | P0DOX7\|IGK | IgK Light Chain | 7 | 1.26 | 0.15 | P0DOX7\|IGK | IgK Light Chain | 7 | 1.26 | 0.15 |
|  | P02787\|TRFE | Serotransferrin | 8 | 0.85 | 0.12 | P02787\|TRFE | Serotransferrin | 8 | 0.95 | 0.65 |
| Significant Proteins | P02774\|VTDB | Vitamin D Binding Protein** | 28 | 0.47 | 0.04 | **P02741\|CRP** | **C-Reactive Protein*** | **158** | **0.48** | **0.05** |
|  | P08582\|TRFM | Melotransferrin | 91 | 0.38 | 0.03 | P0DJI8\|SAA1 | Serum amyloid A-1 protein | 160 | 0.18 | 0.04 |
|  | **P02741\|CRP** | **C-Reactive Protein*** | **158** | **0.45** | **0.03** | P0DJI9\|SAA2 | Serum amyloid A-2 protein | 218 | 0.15 | 0.04 |
|  | P59047\|NALP5 | NACHT | 199 | 0.4 | 0.01 | O94911\|ABCA8 | ABC-type organic anion transporter | 226 | 2.03 | 0.03 |
|  | P20936\|RASA1 | Ras GTPase-activating protein 1 | 239 | 0.41 | 0.04 | Q6MZM0\|HPHL1 | Ferroxidase HEPHL1 | 245 | 0.49 | 0.01 |
|  | The 8 most abundance proteins from our MS analyses are displayed with their associated fold change and p-value (comparing RA vs. non-RA and HPR vs. LPR). None of the top 8 proteins are significant in either analysis. Fold change is dividing RA abundance by non-RA abundance and LPR abundance by HPR abundance.  *P02741\|CRP_HUMAN (C-reactive protein) is a known RA biomarker  **Vitamin D is known to be associated with RA | | | | | Q9NXG0\|CNTLN | Centlein | 254 | 0.17 | 0.04 |
|  |  |  |  |  |  | O43395\|PRPF3 | U4/U6 small nuclear ribonucleoprotein Prp3 | 260 | 0.18 | 0.04 |
|  |  |  |  |  |  | P13797\|PLST | Plastin-3 | 260 | 0.18 | 0.04 |
|  |  |  |  |  |  | P50993\|AT1A2 | Sodium/potassium-transporting ATPase subunit alpha-2 | 260 | 0.18 | 0.04 |
|  |  |  |  |  |  | Q14789\|GOGB1 | Golgin subfamily B member 1 | 260 | 0.18 | 0.04 |
|  |  |  |  |  |  | P09238\|MMP10 | Stromelysin-2 | 289 | 2.12 | 0.03 |
|  |  |  |  |  |  | P01699\|LV144 | Immunoglobulin lambda variable 1-44 | 306 | 0.35 | 0.04 |
|  |  |  |  |  |  | Q6PGN9\|PSRC1 | Proline/serine-rich coiled-coil protein 1 | 316 | 0.33 | 0.05 |
|  |  |  |  |  |  | Q9HCM3\|K1549 | UPF0606 protein | 318 | 0.39 | 0.04 |
|  |  |  |  |  |  | Q15459\|SF3A1 | Splicing factor 3A subunit 1 | 347 | 0.18 | 0.05 |
|  |  |  |  |  |  | P12259\|FA5 | Coagulation factor V | 355 | 30.39 | 0.05 |

# Supplemental Figure 3: HDC simulation

**
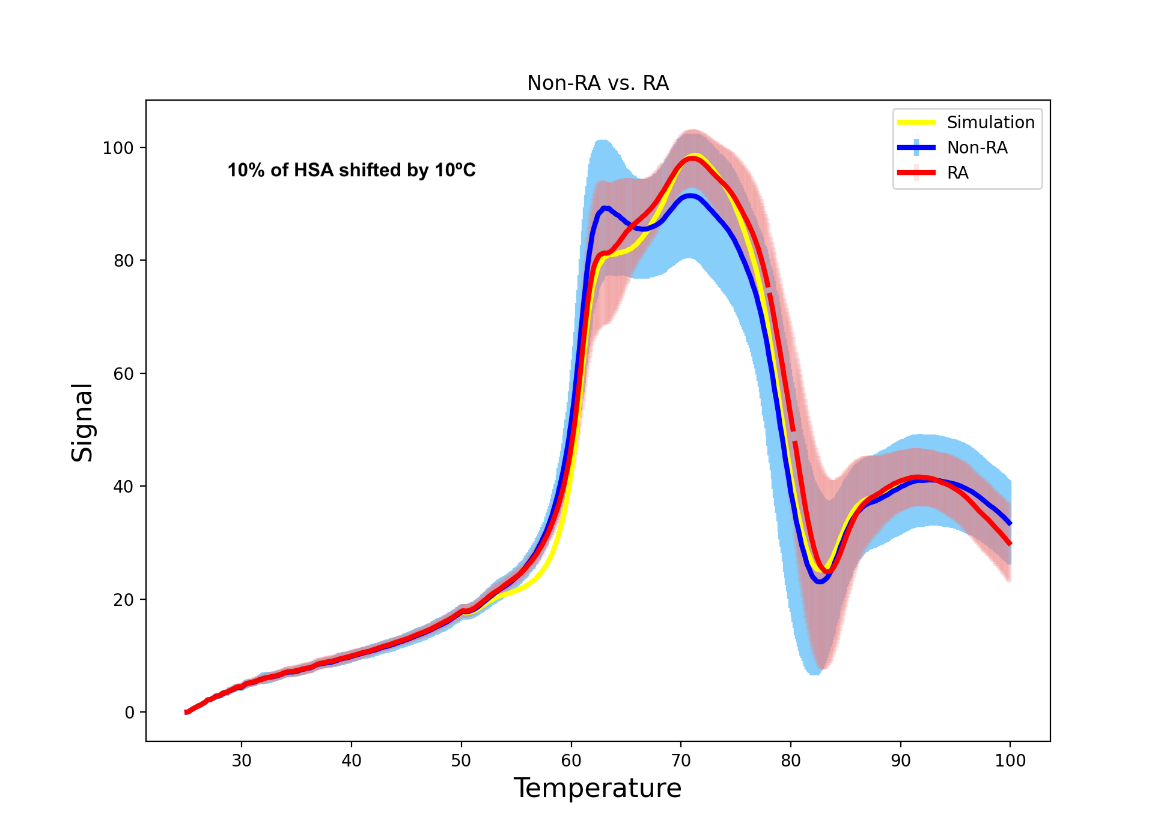
**

Supplemental Figure 3 uses the individually measured HDC of the top 8 proteins in blood serum (from published literature data), simulations were run to find what type of shift would best fit the difference seen between the HDC in RA and non-RA samples (black trace in Figure 3A). The best statistical fit was found to be a 10ºC shift in 10% of the HSA. The "simulated" HDC after the non-RA trace underwent this theoretical shift is shown by the yellow trace here. Among all of the tested "simulations" (different percentages of the top 8 proteins being shifted by various temperatures), this simulated shift aligned the yellow trace most accurately with the RA trace. Visual analysis of this plot indicates that it is also likely that a small percentage of HSA is also shifted by a wider temperature range (some by ~5ºC and some by ~15ºC).

# MS2 spectrum for PTM on HSA

This section includes a representative MS2 spectrum for each of the 41 AEBSF sites and the nine other top PTMs: Dehydration, Hexose, Deamidation, Iodination, Oxidation, Citrulline, Formylation, Amidation, and Di-iodination.

## AEBSF modification Site

### K36


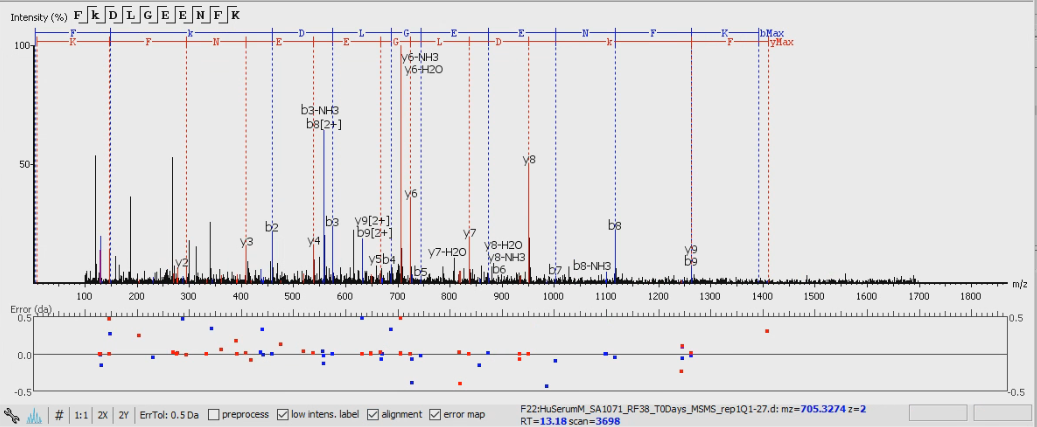


### K75


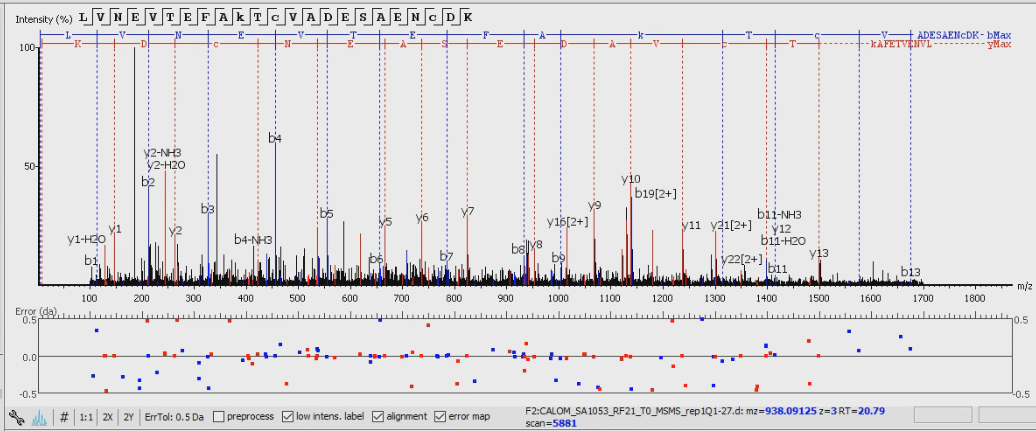


### Y108


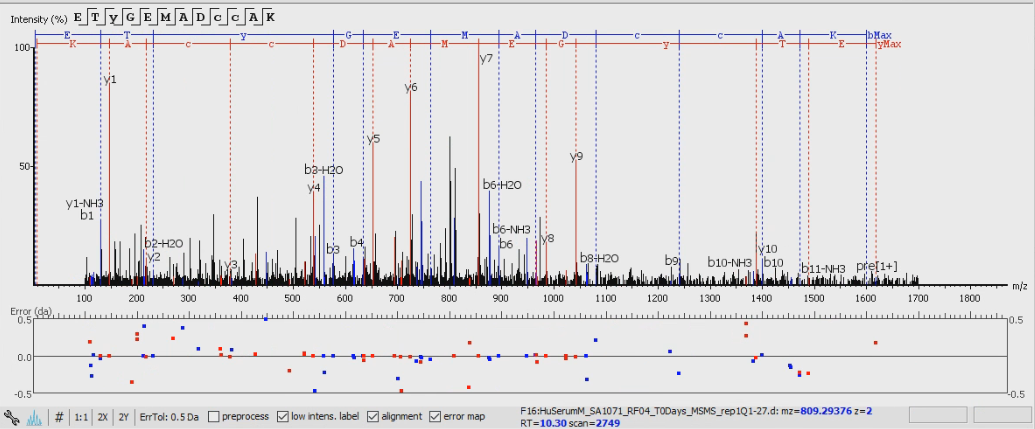


### K117


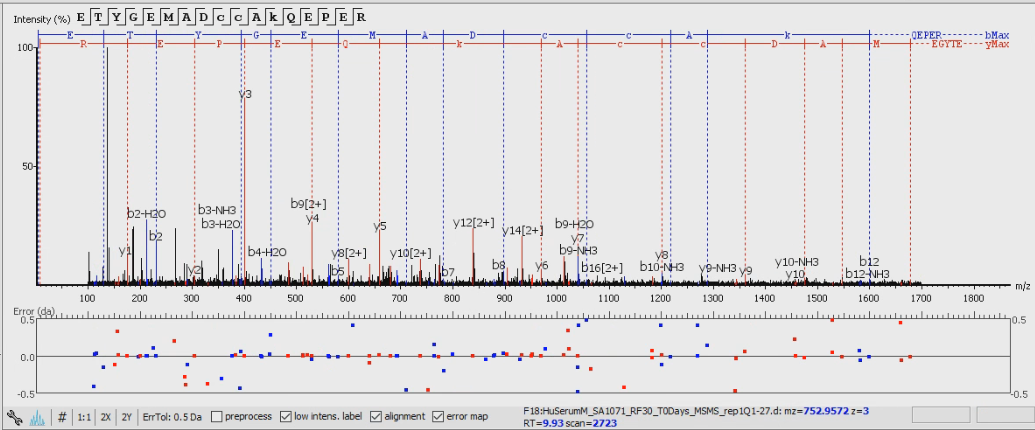


### K160


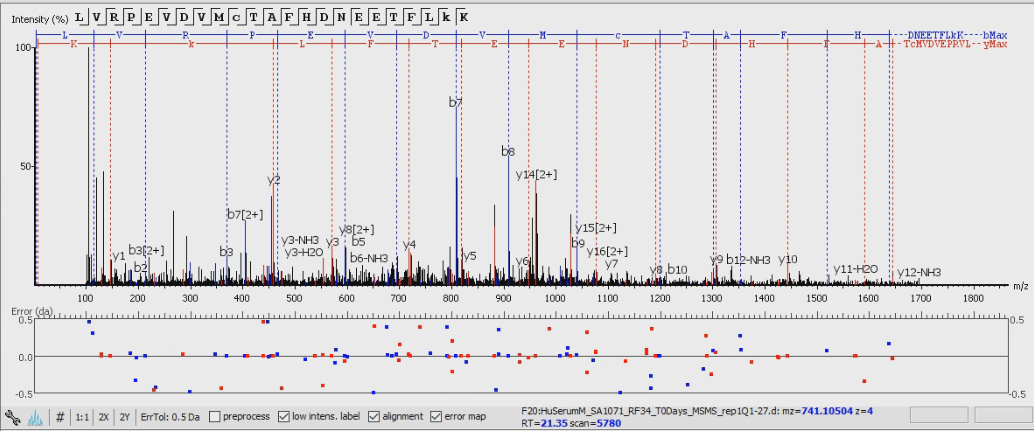


### Y162


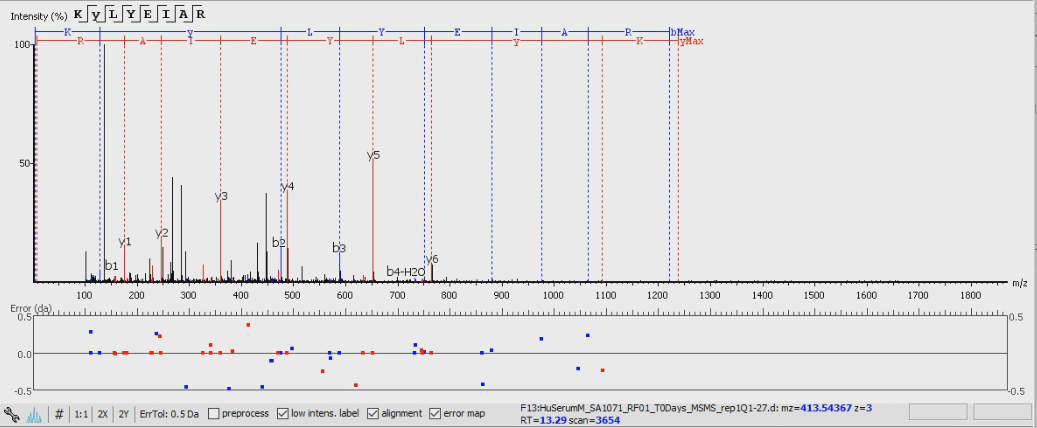


### Y164


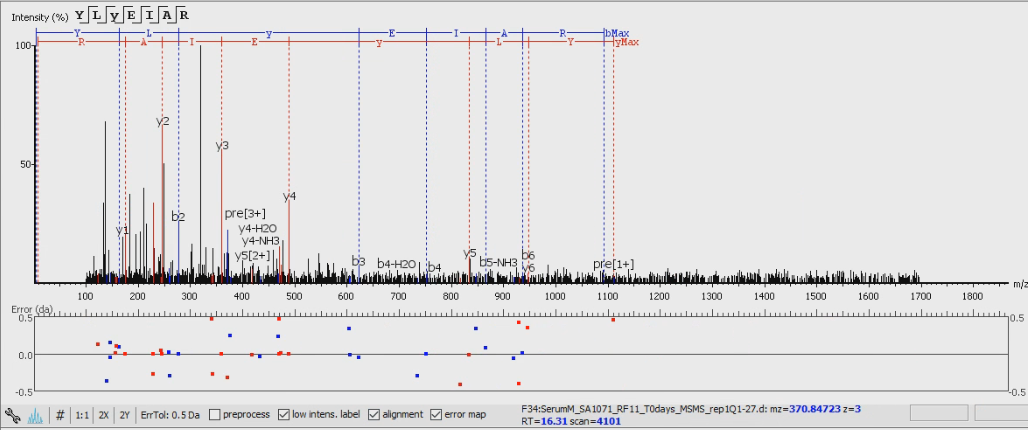
Y185


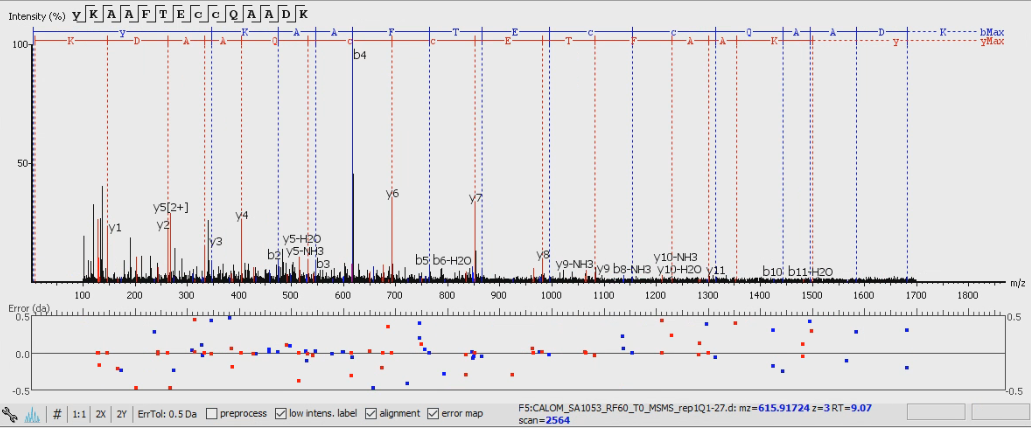


### K205


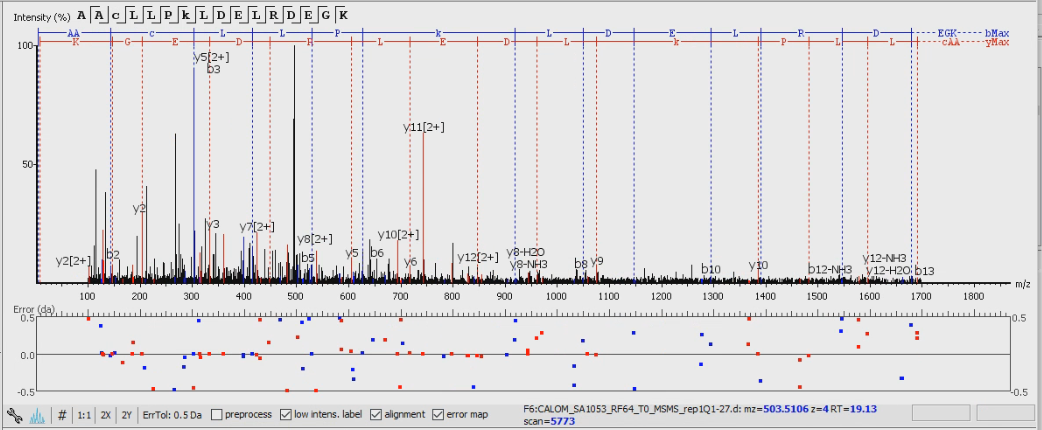


### K214


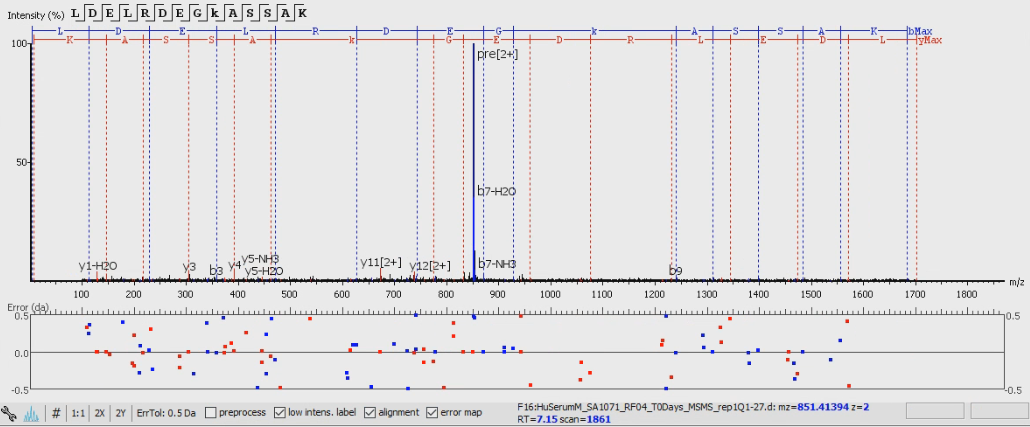


### K223


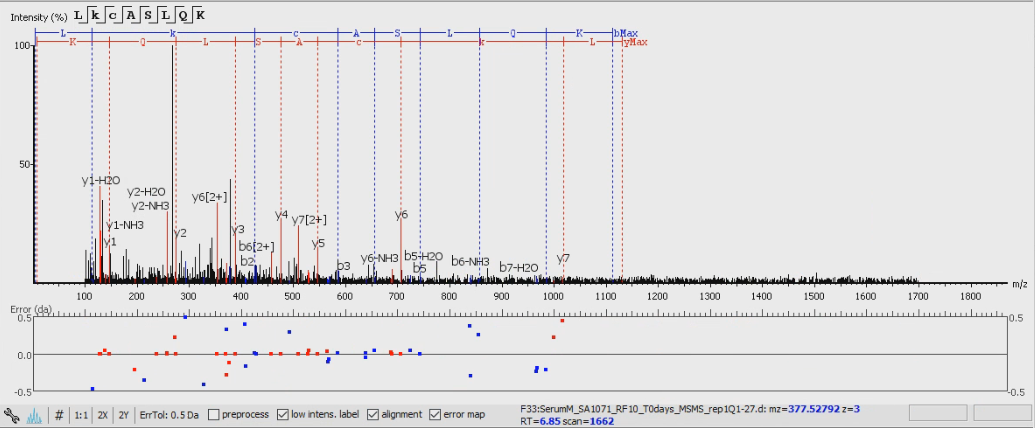


### K229


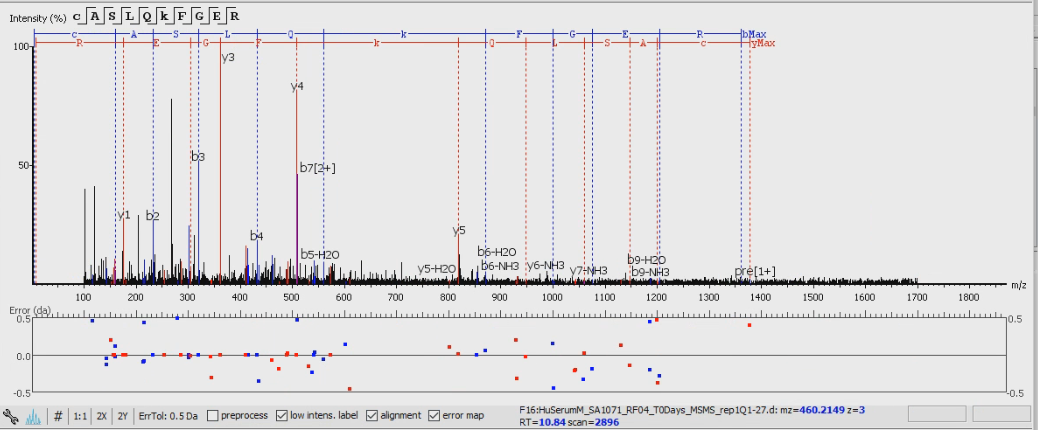


### K236


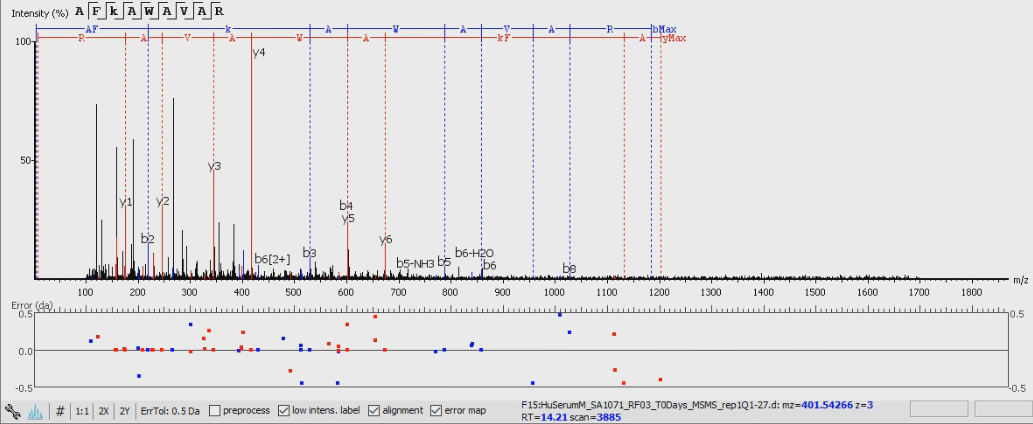


### K249


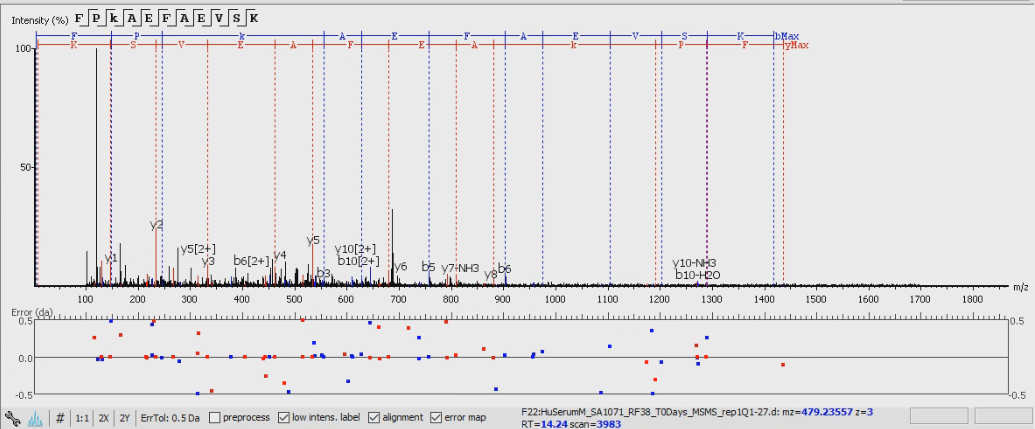


### S256


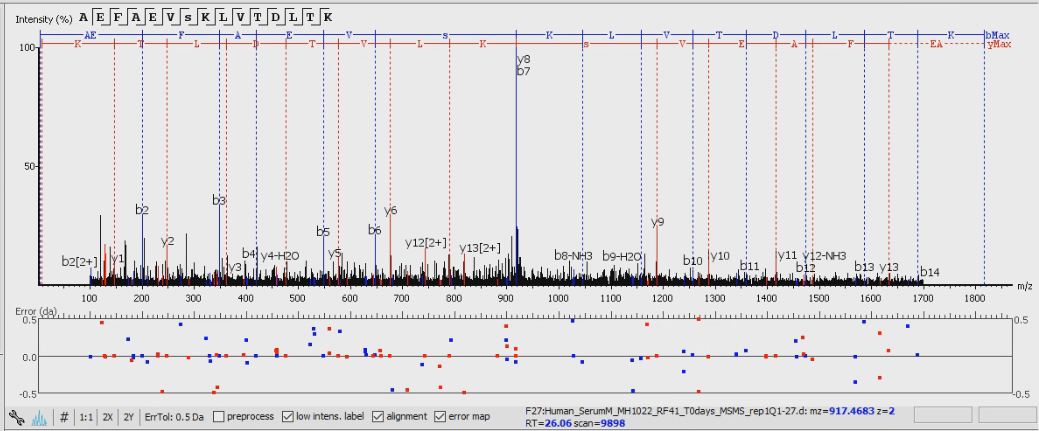


### K257


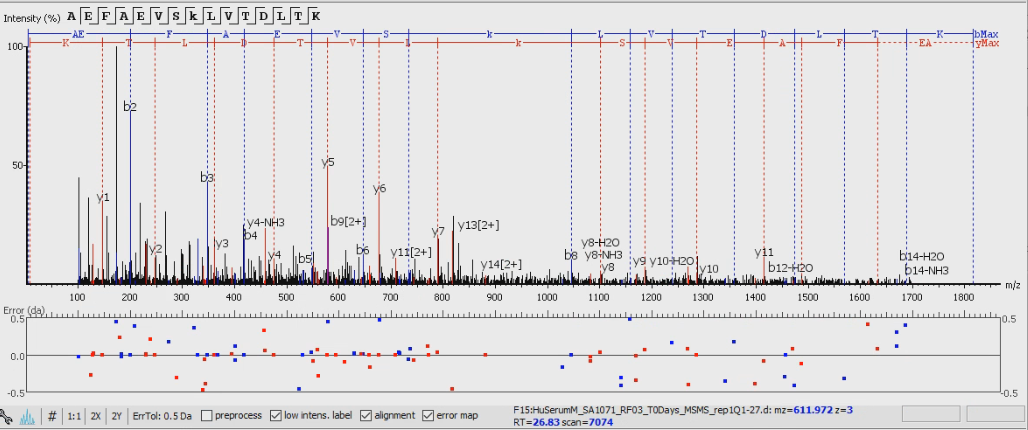


### K286


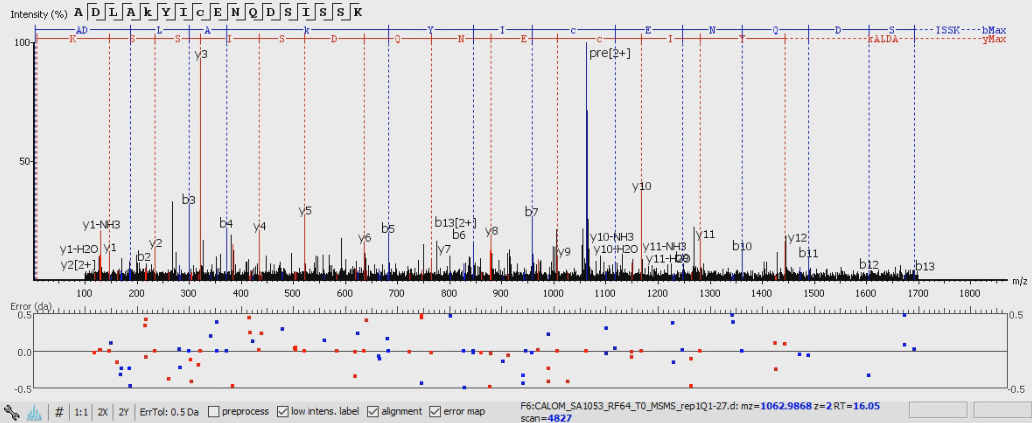


### Y287


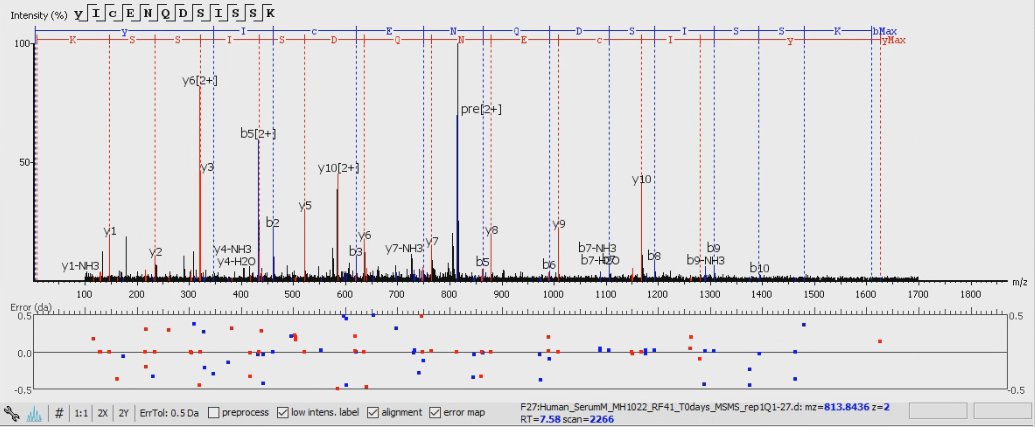


### K305


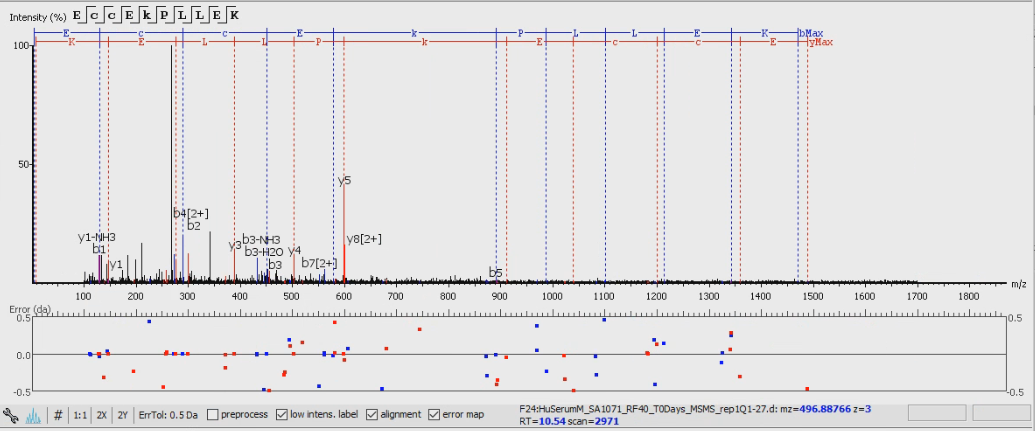


### S311


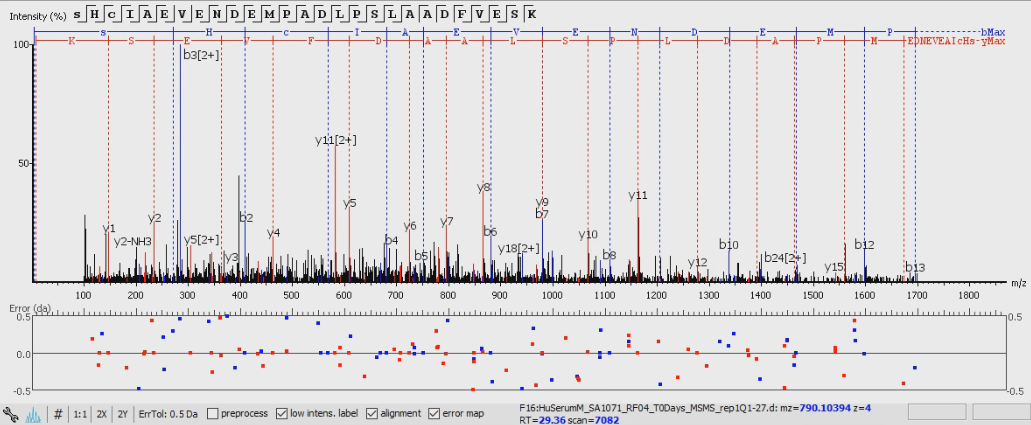


### H312


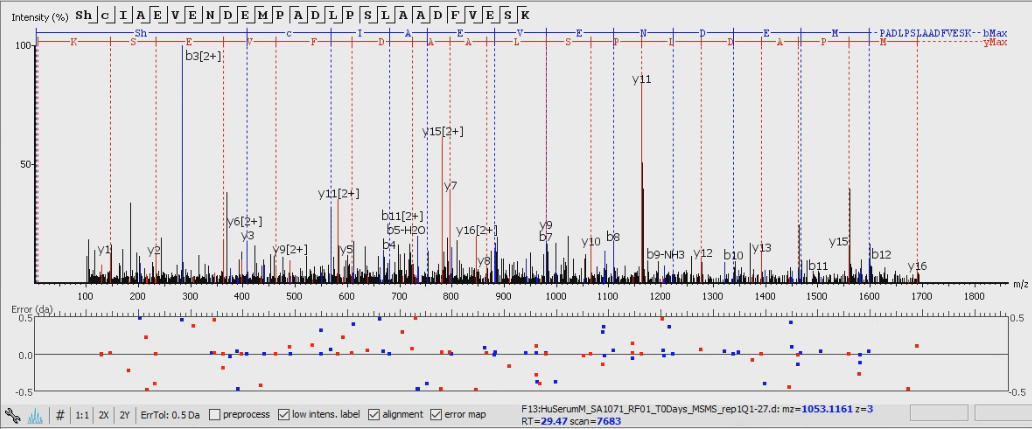


### K337


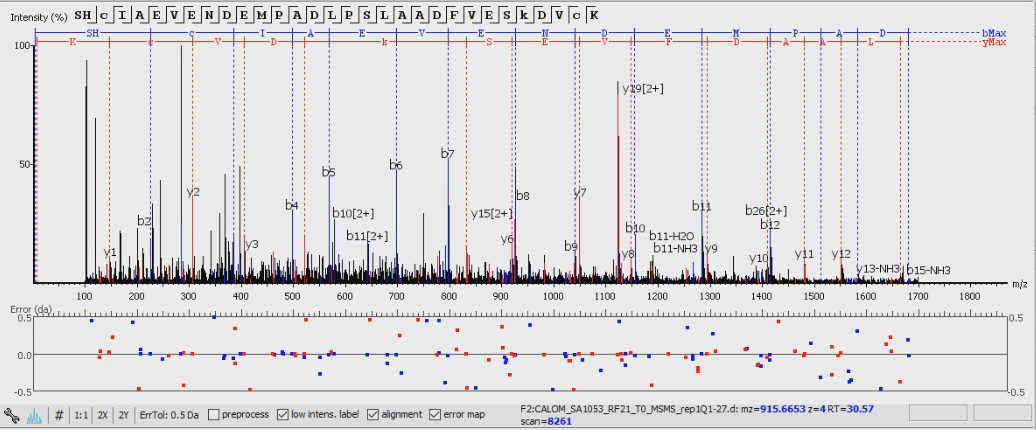


### K375


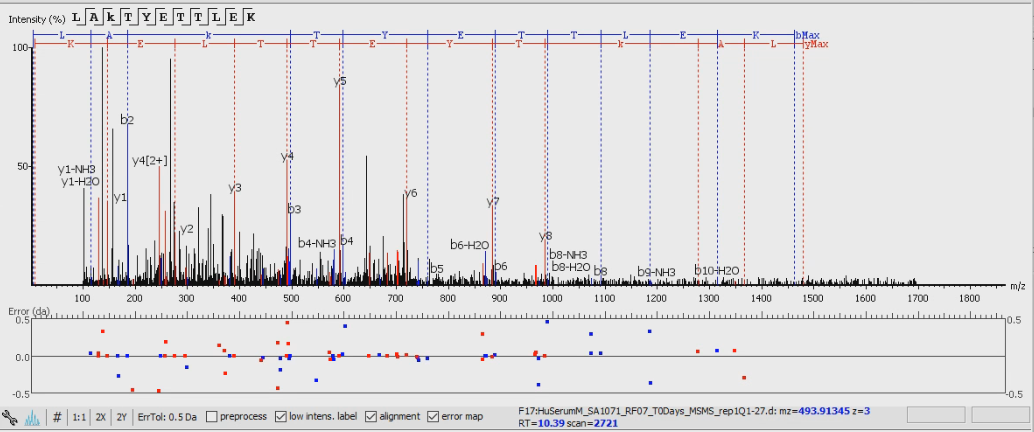


### Y377


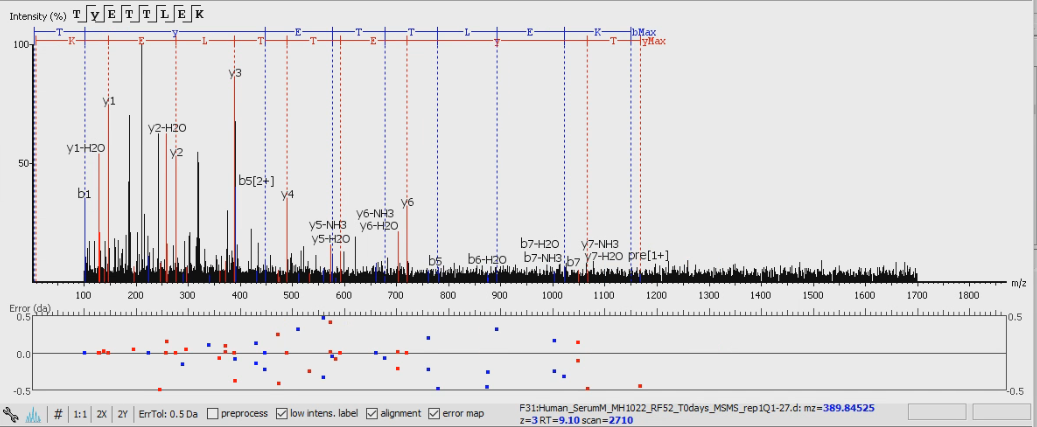


### K383


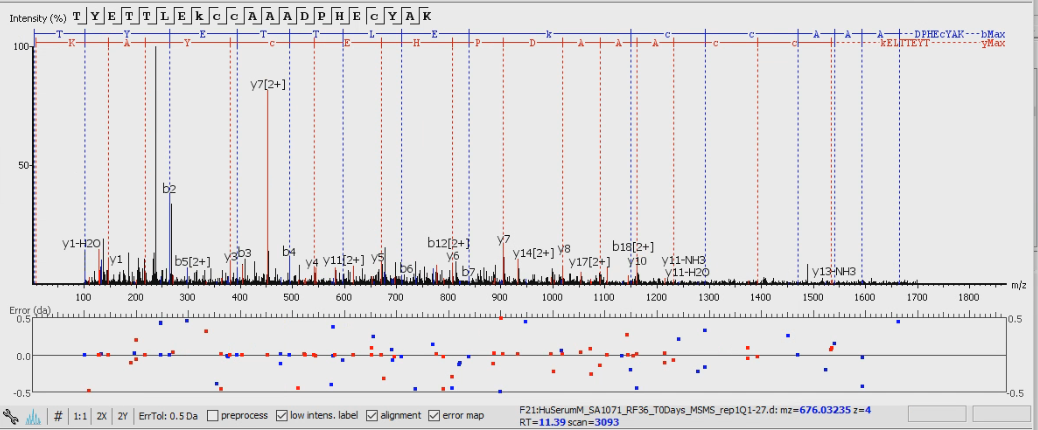


### H391


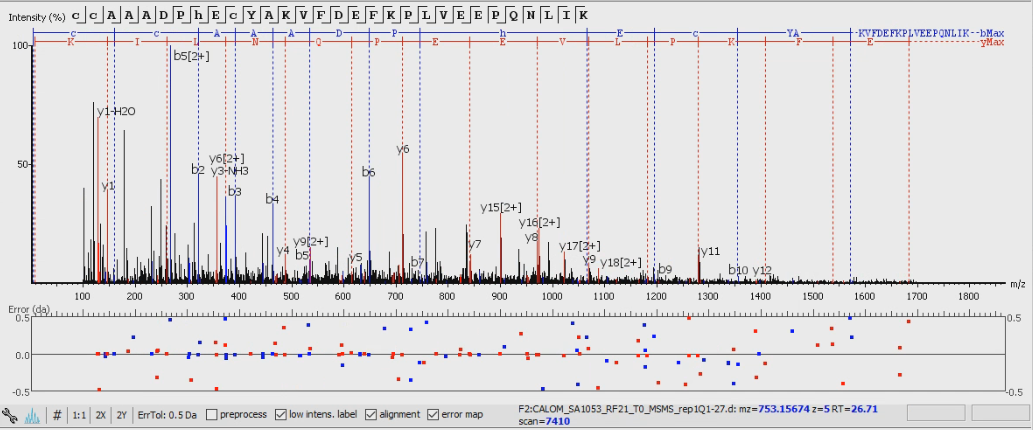


### K402


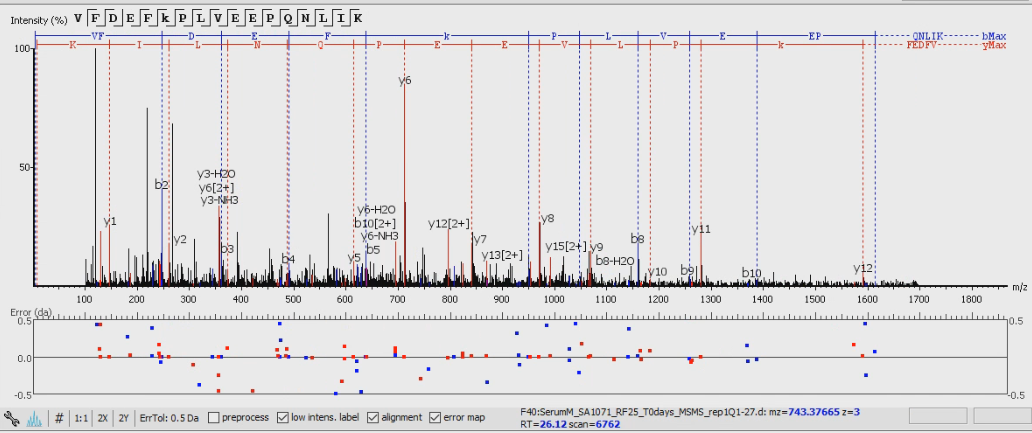


### K413


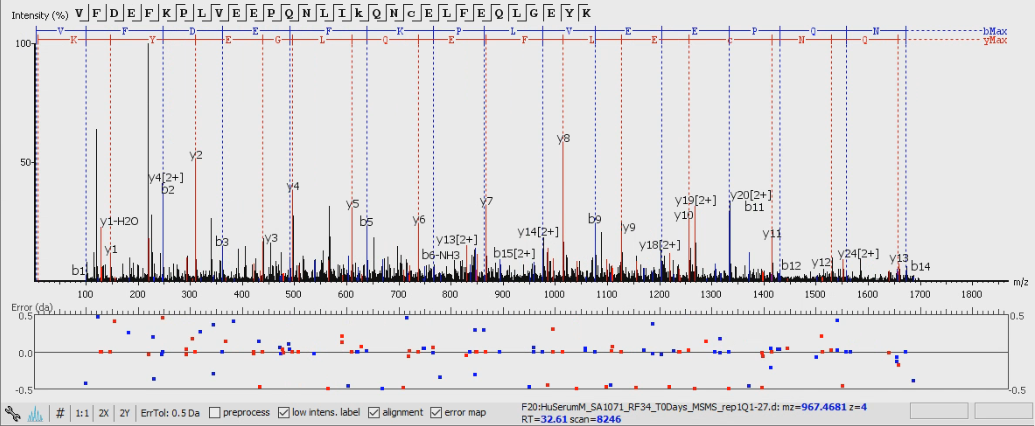


### Y425


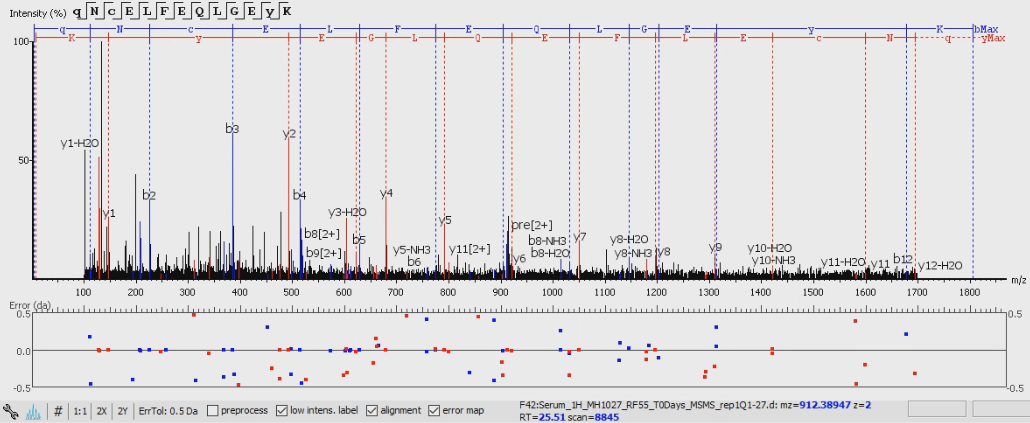


### K426


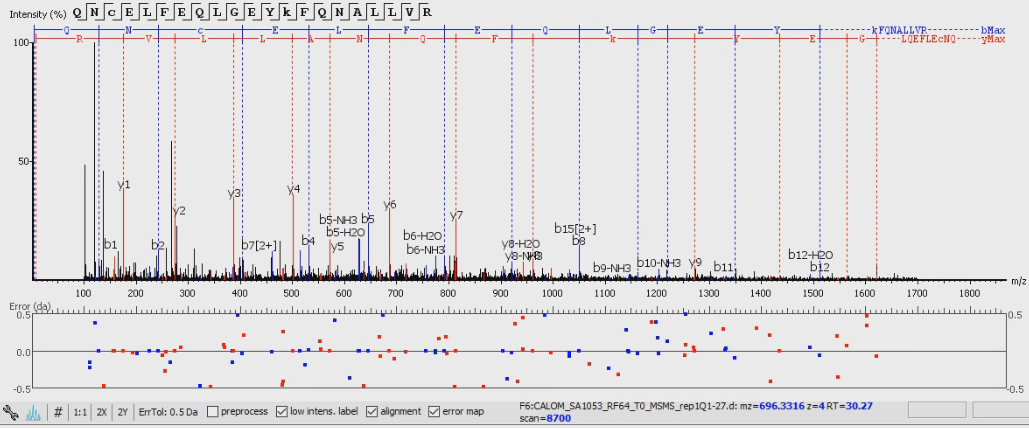


### K456


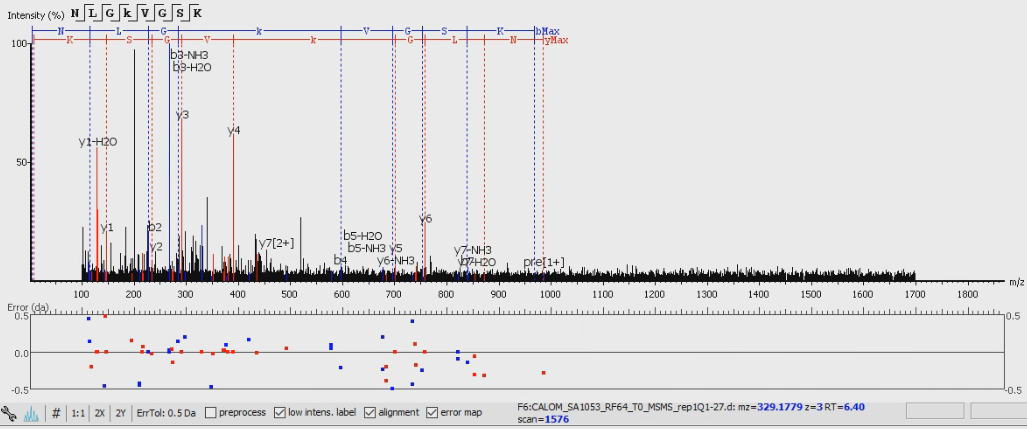


### Y476


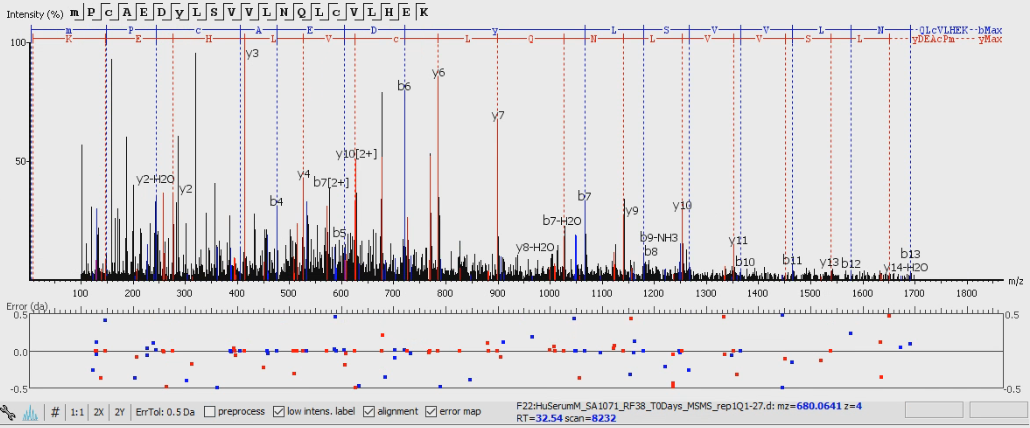


### K499


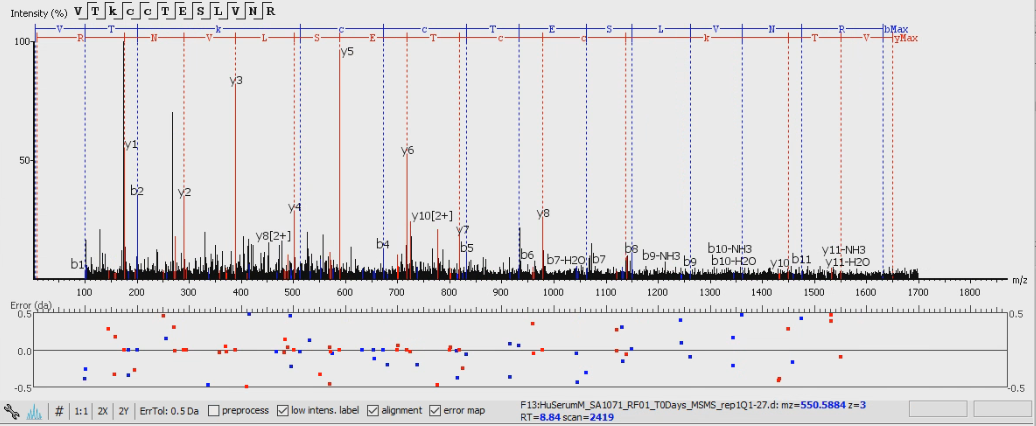


### Y521


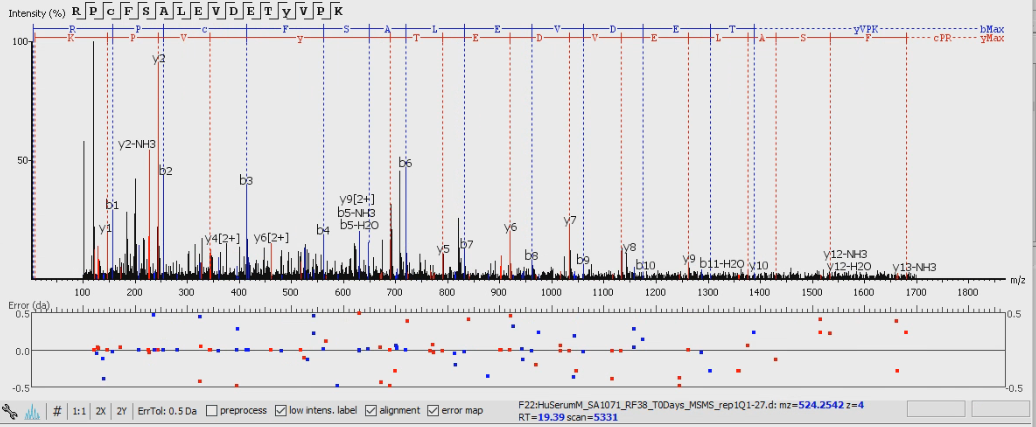


### K543


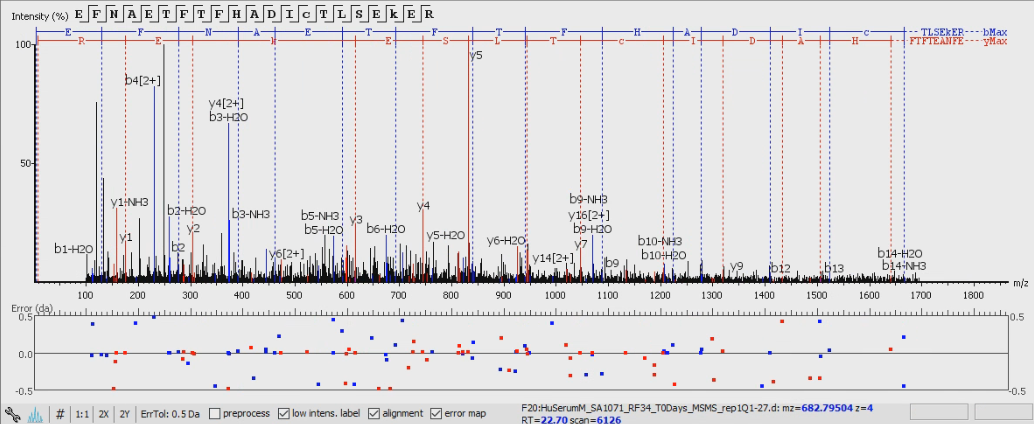


### K565


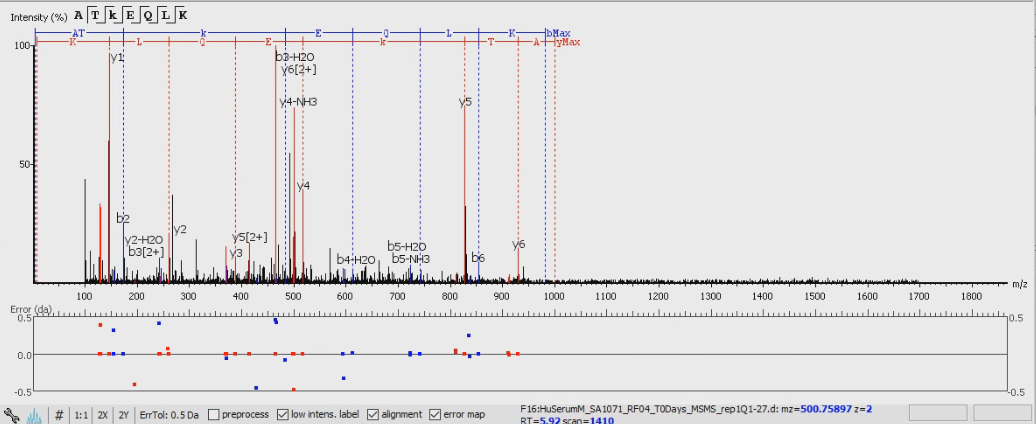


### K569


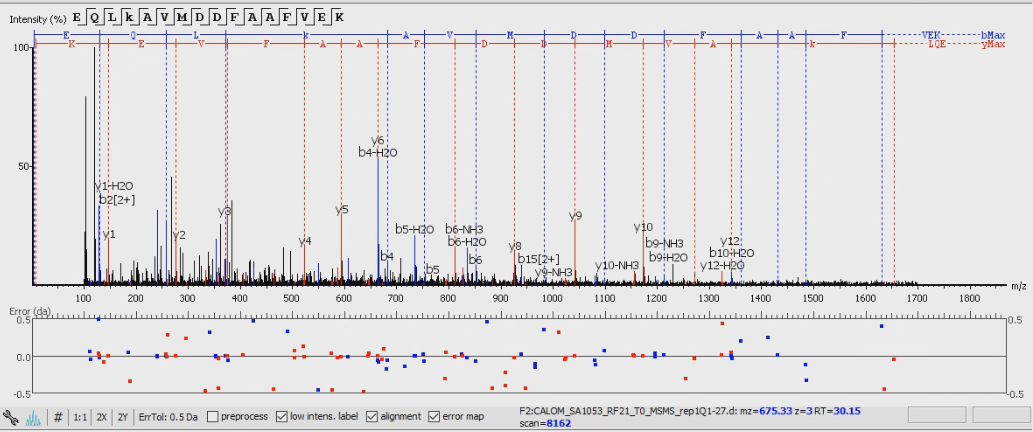


### K581


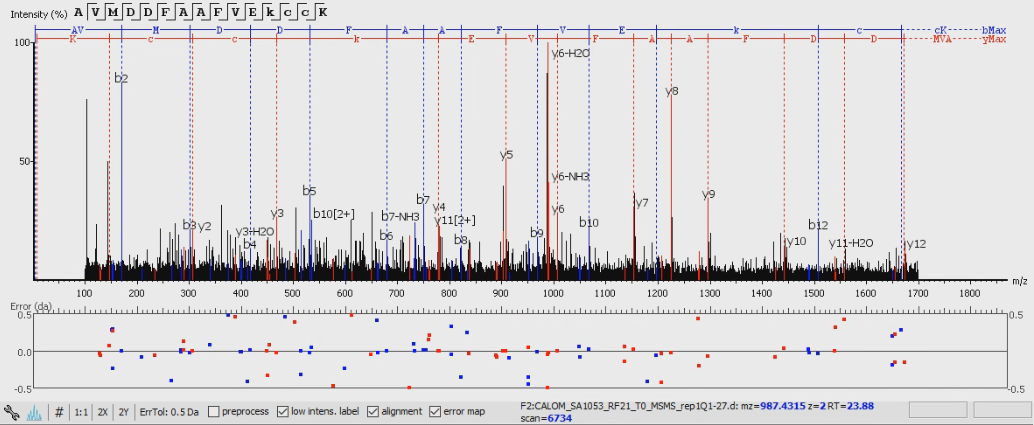


### K584


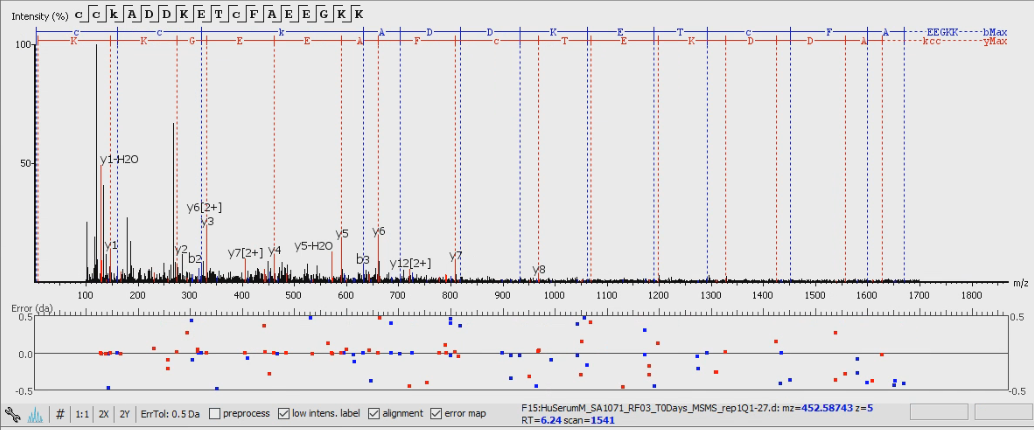


### K588


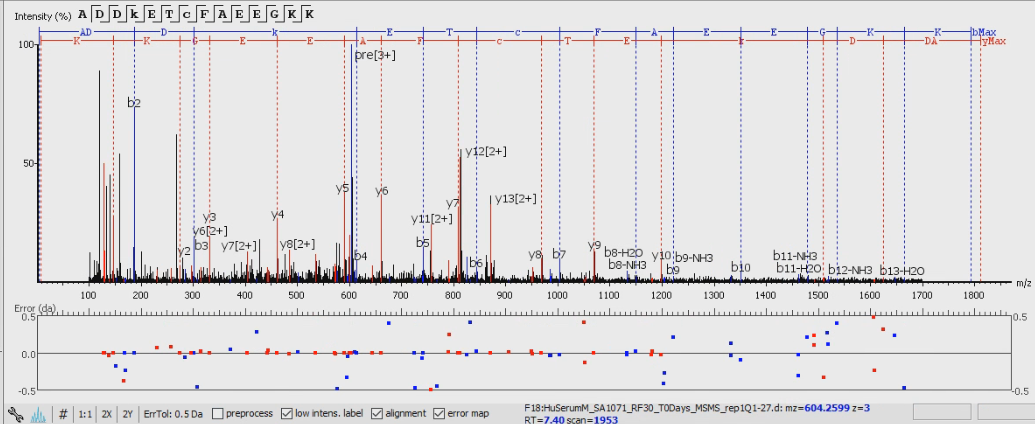


### K597


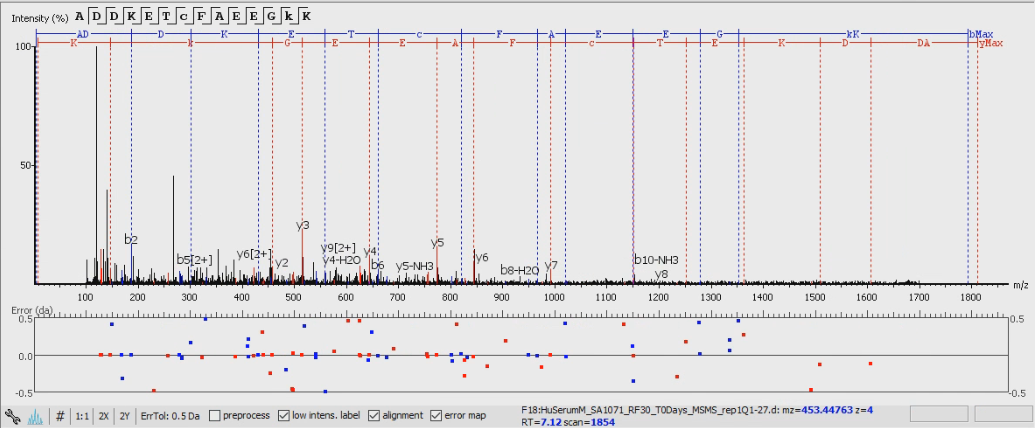


## Dehydration


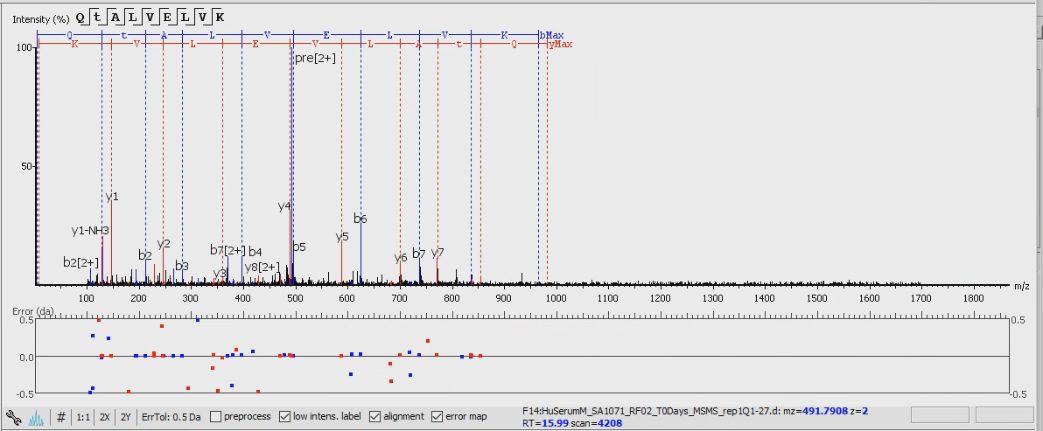


## Hexose


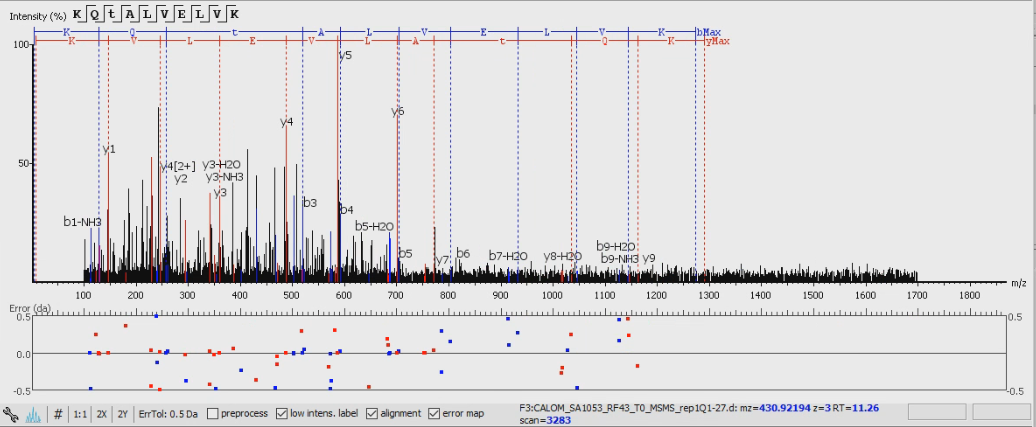


## Deamidation


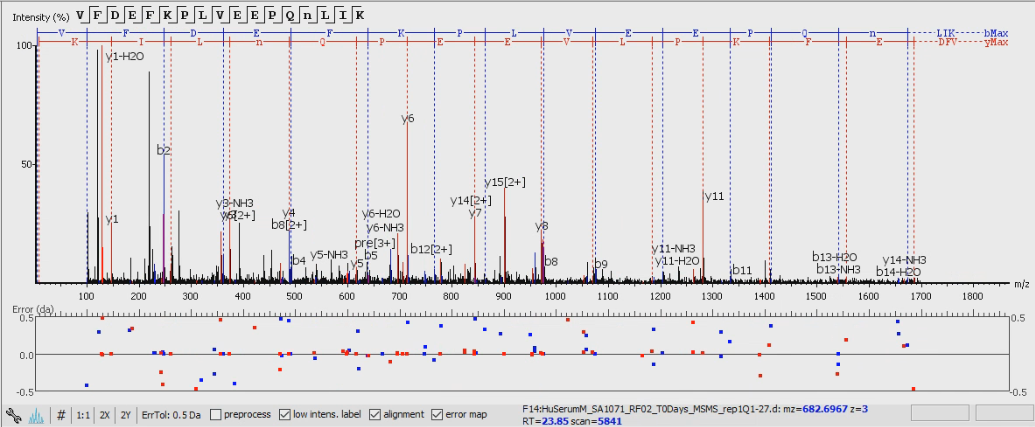


## Iodination


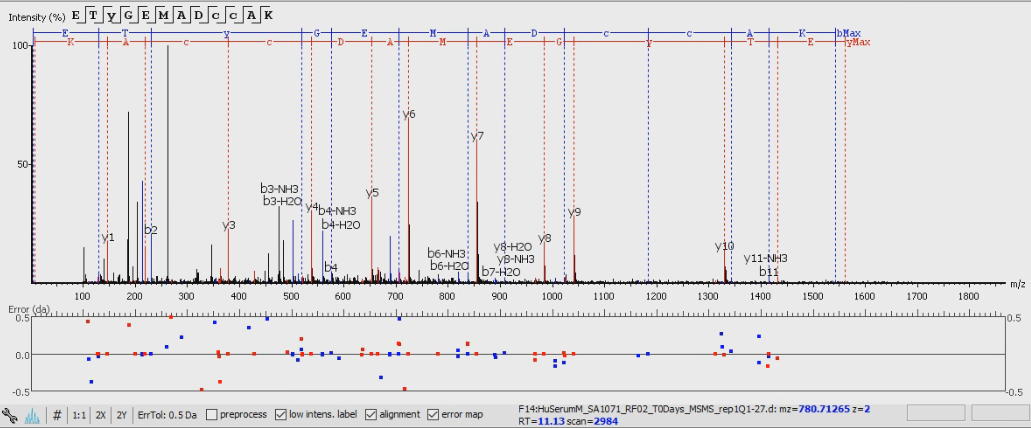


## Oxidation


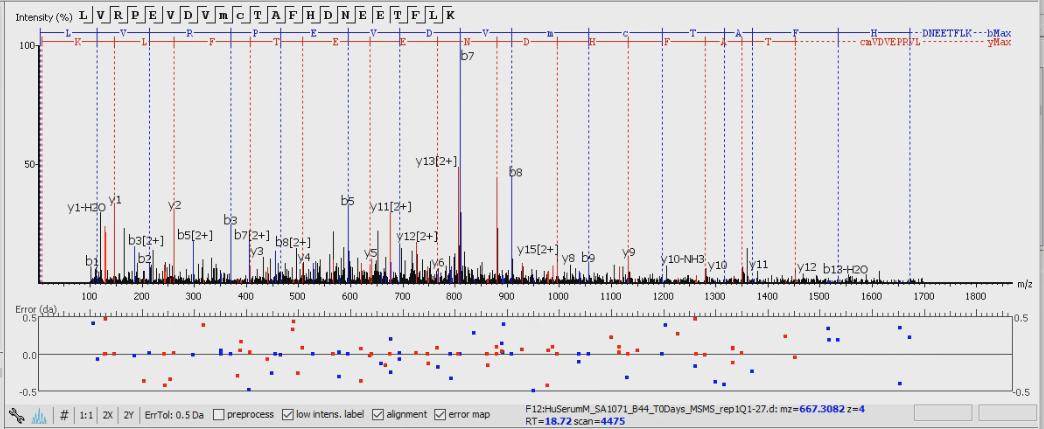


## Citrulline


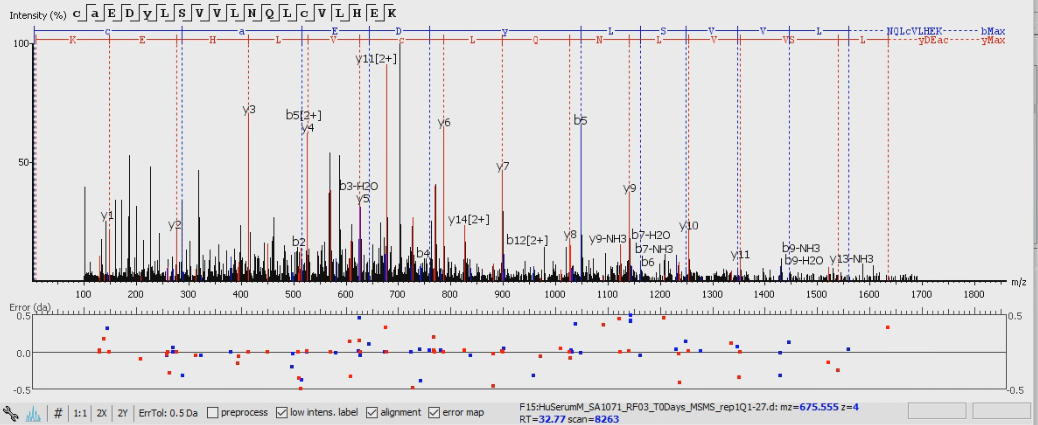


## Formylation


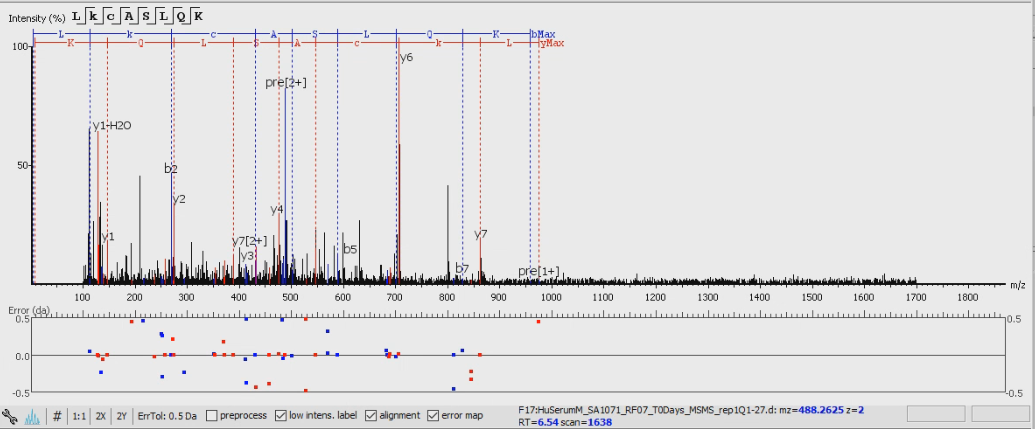


## Amidation


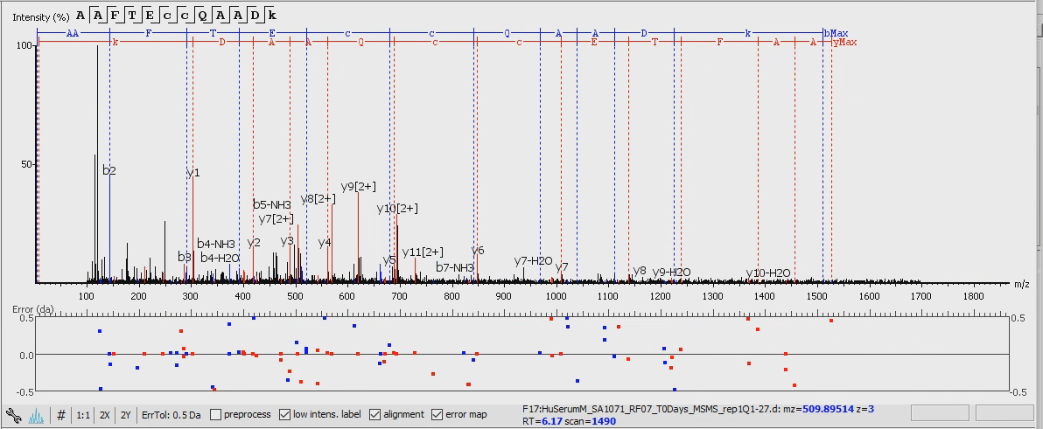


## Di-iodination


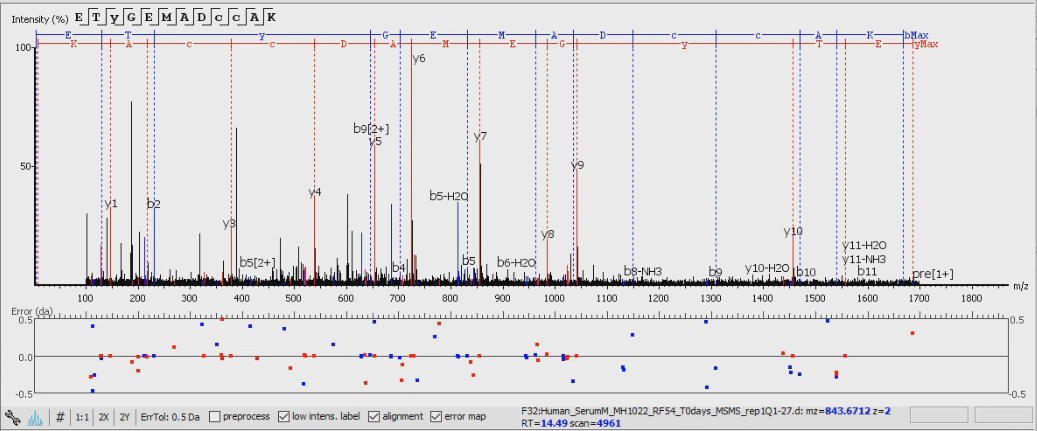

Supplement: S1 File — (DOCX) [file pone.0271008.s001.docx]
